# Sestamibi (99mTc) scan as a single localization modality in primary hyperparathyroidism and factors impacting its accuracy

**DOI:** 10.4103/0972-3919.63591

**Published:** 2010

**Authors:** Yousof Alabdulkarim, Edgard Nassif

**Affiliations:** Department of Surgical Oncology, Notre-Dame Hospital CHUM, Montreal, University of Montreal Qc, Canada

**Keywords:** Localization, primary hyperparathyroidism, sestamibi (99mTc) scan

## Abstract

**Background:**

The proper localization of a hypersecreting parathyroid gland is of vital importance for successful unilateral neck exploration (UNE) and parathyroidectomy.

**Aim:**

In this study we aim to evaluate the (99mTc) sestamibi parathyroid scan as a single localizing modality, and we also assess its relation to the weight of the gland and to the preoperative parathyroid hormone (PTH) levels.

**Patients and Methods:**

We reviewed 170 patients from our hospital (Notre-Dame hospital) from 2005 to 2008, with a mean age of 56.6 years and a female to male ratio of 3.3:1. With primary hyperparathyroidism, all of them had (99mTc) sestamibi parathyroid scan for the localization of the parathyroid adenoma. Preoperative and postoperative PTH levels were recorded. The histopathology reports confirmed the diagnosis and weight of the diseased gland, which were recorded every time. The results were analyzed and correlated with the sestamibi results, to evaluate its accuracy.

**Results:**

Seventy-eight patients (41%) of the 170 had an exact match (EM) sestamibi results, 81 (51.6%) had a partial match, and only 11 patients were reported as mismatch. Analyzing the mean weight of the gland in each group between matching (EM, PM) versus mismatch resulted in a mean difference of 0.823 g (1.05 and 0.247 g, respectively) *P* = 0.045. Hyperplasia to adenoma ratio was more in the partial matching group (18.5%) versus the exact matching group (7.6%). Finally the mean PTH level was higher in the EM group (28.8 pmol/L) compared to the mismatch group (10.1 pmol/L) *P* = 0.02. Overall sensitivity and specificity for the (99 mTc) sestamibi in our data was 98.1 and 97%, respectively.

**Conclusion:**

(99mTc) sestamibi is a highly accurate test that can be employed as a single localizing modality for identifying a hypersecreting parathyroid, a UNE, or a parathyroidectomy. The weight of the gland plays an important role in the accuracy of the test, as also the preoperative PTH levels.

## INTRODUCTION

Despite the famous statement that "the best localization technique for parathyroid is a capable surgeon", many clinical trials were conducted to evaluate and compare the different modalities, to come up with the best protocol that is not only accurate, but also relatively easy and cost effective.[1‐3]

Preoperative and perioperative radiological[4] and nuclear medicine studies, either individual or in combination, of intraoperative bilateral and unilateral neck exploration[5] and four gland sampling, PTH venous sampling, minimal invasive radiological guided parathyroidectomy,[6,7] even frozen section,[8] have been evaluated[9] extensively in literature.

In this study we evaluate the preoperative Tc99m-sestamibi scintigraphy as a single localization modality that is not only easy to perform, but is also a cost effective[10] technique, and is not operator-dependent.[11,12].

Few reports tried to emphasize on the factors affecting the accuracy of a sestamibi scan for parathyroid, many of which talked about the various technical modifications that might enhance the accuracy, and some concentrated more on the characteristics of the diseased gland and its relation to the precise results obtained from the sestamibi scan.

We tried to study the relation between the histopathology findings, in terms of type of abnormality (hyperplasia vs. adenoma), physical characteristics of the hypersecreting gland, including the weight of the gland, as well as the relation to the parathyroid hormone obtained preoperatively, and the overall results of the successful localization of the parathyroid gland intraoperatively in a UNE and parathyroidectomy.

## PATIENTS AND METHODS

From January 2005 to July 2008, a series of 170 consecutive patients (mean age ± SD) 56.6 ± 13.1 years, with a female to male ratio of 3.3:1 from the hospital Notre-Dame (one of the central hospitals of the university of Montreal).

Ionized calcium level of 1.42 ± 0.14 mmol/L, Mean PTH level preoperative 53.4 ± 88 pmol/L range 0.2 – 530 pmol/L.

All patients had a normal renal function; and they were operated by the same surgeon. Preoperatively they all had Tc99m-sestamibi scintigraphy as a localization technique.

Inclusion criteria were primary hyperparathyroidism, presence of sestamibi scan regardless of results, all patients had to be treated surgically, and confirmation of a hyperfunctional parathyroid gland by histopathology.

The exclusion criteria included any patient with abnormal renal function, inability to retrieve the sestamibi scan (either not done, missing, or refused by the patient), any concomitant thyroid surgery in the same setting.

The technique employed was a standard double isotope Tc99m-sestamibi scintigraphy and I^123^, with a neck window followed by a mediastinal thoracic window.

The confirmatory rPTH was drawn in the recovery room instead of immediately post excision of the hyperfunctional gland. We never use the frozen section intraoperatively for parathyroidectomy in our institute.

Follow-up was done with the collaboration of the Department of Endocrinology and each patient was followed up in our Outpatient Clinic at least once post discharge from the hospital, and ionized calcium and PTH results were followed for an average of 18 months (range between three months and two years).

On the basis of the histopathology results, biochemical improvement, and clinical findings, we analyzed each patient with the Tc99m-sestamibi scintigraphy results, in order to detect any relation between these variables against the accuracy of the Tc99m-sestamibi scintigraphy, and accordingly the patients were divided into three subgroups:

Exact matching (EM): where the Tc99m-sestamibi scintigraphy scan results were accurate for the site (upper or lower) as well as for the side (right or left) and for the number of hyperfunctional gland(s).

Partial match (PM): where the results of the scan were accurate only for either side (right or left) or site (upper or lower).

Mismatch group (MM): where the results were not corresponding to the exact location of the hyperfunctional gland - which was identified later by pathology reports - or were not accurate for the number of diseased gland(s).

### Statistics

Data were collected utilizing Microsoft Excel^®^ 2003, they were analyzed using SPSS for windows^®^ 14.0. We expressed our results in Mean ± SD.

The comparison of results was performed by *t*-test and chi-square tests; the curves were designed based on scattered points, to compare paired values whenever needed. The final results were considered statistically significant when the two-tailed *P* value was <0.05.

A literature review was performed using PubMed and MDConsult with Key words including sestamibi parathyroid scan, primary hyperparathyroidism, and preoperative localization of parathyroid adenoma, for English articles published between 1980 and 2008. Studies for secondary hyperparathyroidism were excluded.

## RESULTS

Starting with 178 patients, eight patients had to be excluded because of lack of data or inability to view the parathyroid sestamibi scan.

**Figure 1:**
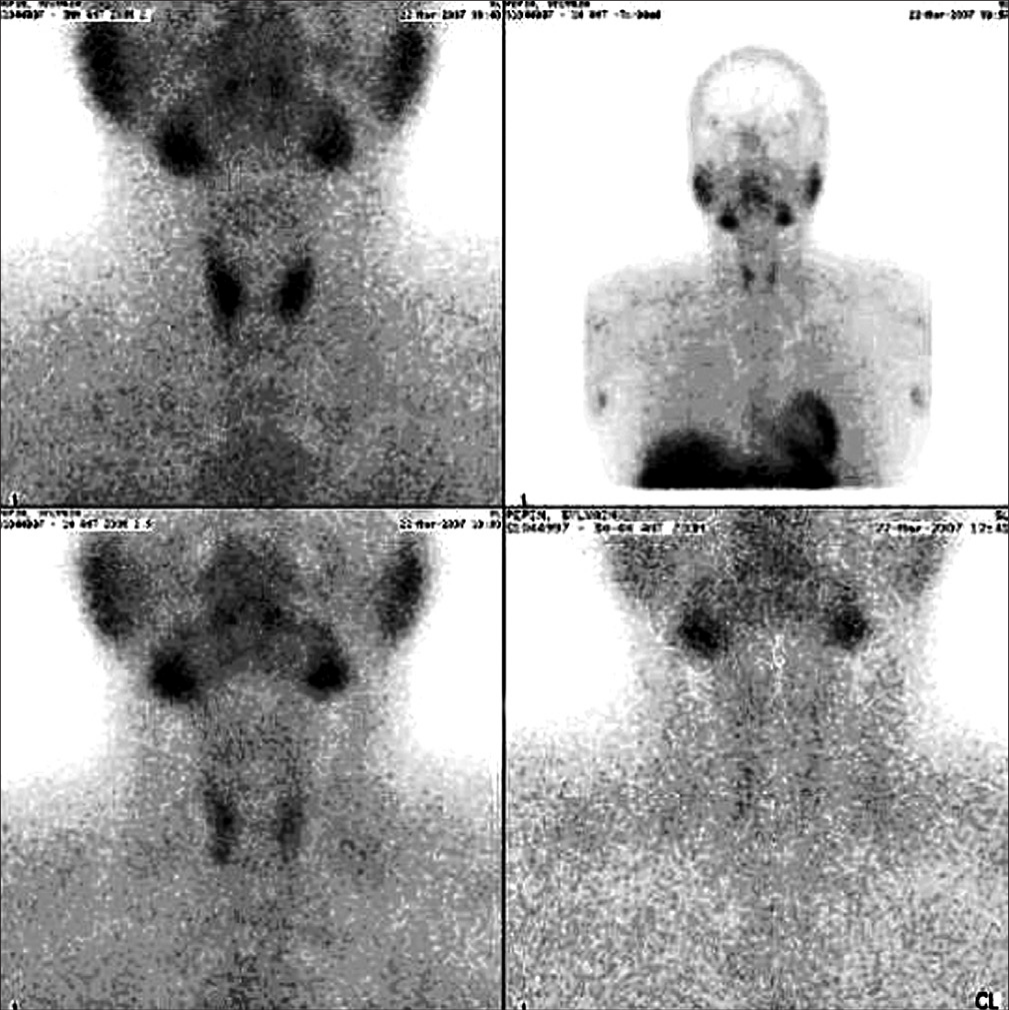
Right Inferior parathyroid adenoma

In the group, 78 patients (41.7%) had an exactly matching sestamibi scan for hyperfunctional gland and underwent a UNE [Figure 1], the success of a complete excision of the parathyroid diseased gland was confirmed by a 50% drop in the preoperative PTH compared to the rPTH in the recovery room 30 min post operation, as well as by the final histopathology results. The patients of this group showed signs of normalization of their parathyroid function biochemically and clinically in the follow-up, in the OPD.

Eighty-one patients (51.6%) had a partially matching sestamibi scan, however, although they still underwent a UNE, intraoperatively the decision had to be made according to the visualization of the diseased gland. and this group also had the same protocol for confirmation and follow-up like those of the EM group.

Only eleven patients had a confirmed mismatch sestamibi result; Eight of the eleven had three-and-a-half parathyroidectomy, and three patients had a double adenoma and were operated again for recurrent symptomatic hyperparathyroidism; all of the three were cured after the second operation.

Therefore, in general the matching scans, either partial or complete, comprised of 159 patients of the 170 subjects (93.5%) versus eleven patients (6.4%). With a sensitivity of 98.1% and a specificity of 97.01%, the positive predictive value was 97.6%, and the false negative and false positive were 3 and 1.8%, respectively.

The weight of the gland ranged between 0.1 g and 10 g, with a mean of 0.88 g. Among the group of EM the mean weight of the excised gland was 1.06 ± 1.43 g, (ranging between 01 and 10 g), while in the PM the mean was 1.7 ± 2.14 g (ranging between 0.11 – 9.0 g). In the Mismatch group the range of the hyperfunctional gland was between 0.01 – 0.61 g, with a mean of 0.24 ± 0.27 g.

**Figure 2:**
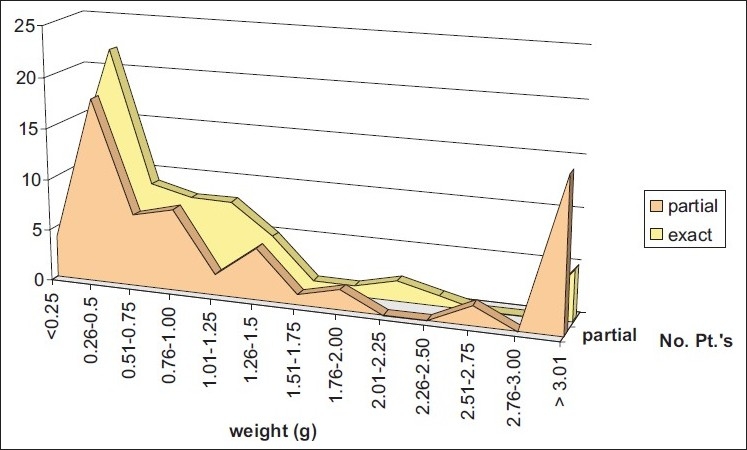
Relation between the weight of the gland in each of the EM and PM subgroups. There was also evidence of a relation pattern between the matching group and the magnitude of the preoperative PTH, when compared to the mismatch group

By comparing the difference, the average weight between the matching group (PM and EM) and the mismatched group was 1.058 g versus 0.247 g, respectively, with a mean difference of 0.823 g (*P* = 0.045) [Figure 2].

In the exactly matching group; six patients (7.6%) of the 81 had a confirmed pathology report of hyperplasia with the remaining 72 having adenoma. The partially matching group showed a higher ratio of hyperplasia; where 15 patients out of the 81 were diagnosed with hyperplasia (18.5%).

## DISCUSSION

Since the introduction of Technetium-99m (99mTc) sestamibi for parathyroid imaging in 1989,[13] it has been widely used for the determination and location of the hyperfunctional parathyroid adenoma, with encouraging results.[14] The technique employed underwent several modifications that resulted in an overall improvement of its sensitivity from 89 to close to 100%, in recent reports.[12,15]

In our study we evaluated the weight of the hyperfunctional parathyroid as a predictive factor for the accuracy of the preoperative Tc99m-sestamibi scintigraphy study; the mean weight of 1.05 ± 0.4 g was associated with a high accuracy rate. Interestingly, we found that these results were accurate for both parathyroid adenoma and hyperplasia. On the other hand, tiny adenomas with an average weight less than 0.600 g were difficult to localize, and were associated with either negative results or a misleading location that required an experienced surgeon to intraoperatively identify the suspicious gland.[16]

The significant statistical difference between the matching and mismatching groups was not surprising; several authors came up with more or less similar findings.[17‐19]

This was probably explained by the correlation between the weight of the gland, its Oxyphil cells content, with their increased sestamibi uptake, and storage by the mitochondrial-rich organelles.[17,20]

However, the absence of a statistically significant difference between the exact and partially matching groups may be explained by two factors; the eventually improved experience and accuracy in the selected institute, with the gradual increase in the number of patients undergoing the procedure every month in the last couple of years; and secondly because of the ratio of pathologically confirmed hyperplasia to adenoma, which was 18.5% in the PM group in comparison to 7.6% in the EM. This has been also reported particularly in glands that weigh less 0.8 g., with a tendency to cause a confusion between the upper and lower glands; rather than the left- and right-sided adenomas.[17]

**Figure 3:**
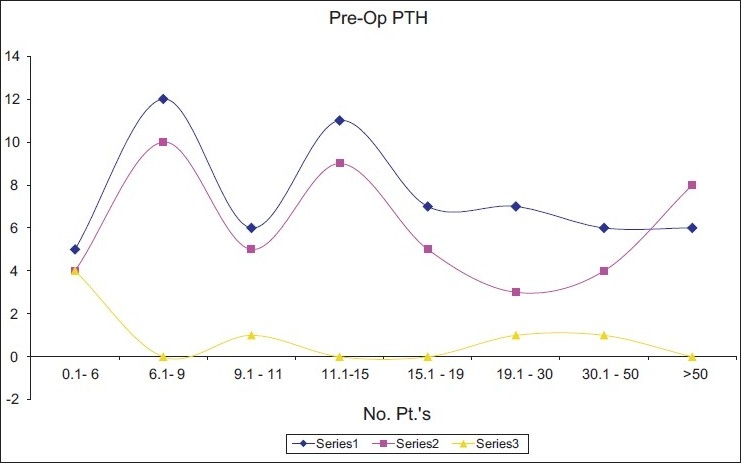
The relation between PTH and the three subgroups of patients. Series 1(EM), series 2 PM, and series 3 MM Preoperative Intact PTH levels in the EM group were expressed by the mean value of 28.8 pmol/L, and on the other hand the mean value of intact PTH for the mismatch group was 10.1 pmol/L, with a *P* value of 0.02.

The magnitude of preoperative PTH was closely related to the accurate results of the (99mTc) sestamibi scan. In our sample of patients the mean value of the preoperative intact PTH in the EM group was 28.8 pmol/L, while the recorded mean PTH level in the mismatch group was 10.1 pmol/L, with a mean difference of 18.6 that resulted in a *P* value = 0.02. That suggested a nonincidental relation between the two [Figure 3].

### Limitations

The challenge that faces one who studies the results of parathyroidectomy is the occasional confusing pathology that cannot differentiate hyperplasia from adenoma.

Variation in the interpretation reports is another factor that affects the final surgical decision, but luckily many of these challenges did not exist among our population of patients.

## CONCLUSION

The routine utilization of Tc99m-sestamibi scintigraphy scan as a single localization test, for identifying a parathyroid adenoma, is an accurate and efficient method that helps to avoid unnecessary bilateral neck exploration, due to its high sensitivity and specificity.

It can be performed as a part of the preoperative assessment with clear cost effectiveness compared to multiple localizing techniques.

The weight of the adenoma plays an important role in the accuracy of this test as well as the preoperative PTH level.

Tiny adenomas tend to have a low weight and are in general associated with lower preoperative PTH values.

## References

[1] Bergert H, Zimmermann T, Ockert D, Alldinger I, Kersting S, Saeger HD (2003). Intraoperative chemiluminometric assay for simplified localization of parathyroid adenomas during surgery for primary hyperparathyroidism. Talanta.

[2] Chen H, Sokoll LJ, Udelsman R (1999). Outpatient minimally invasive parathyroidectomy: A combination of sestamibi-SPECT localization, cervical block anesthesia, and intraoperative parathyroid hormone assay. Surgery.

[3] Hajioff D, Iyngkaran T, Panagamuwa C, Hill D, Stearns MP (2004). Preoperative localization of parathyroid adenomas: Ultrasonography, sestamibi scintigraphy, or both?. Clin Otolaryngol Allied Sci.

[4] Livingston CD, Victor B, Askew R, Abikhalid J, Meynig J, Lindsey M (2008). Surgeon-performed ultrasonography as an adjunct to minimally invasive radio-guided parathyroidectomy in 100 consecutive patients with primary hyperparathyroidism. Endocr Pract.

[5] Westerdahl J, Bergenfelz A (2007). Unilateral versus bilateral neck exploration for primary hyperparathyroidism: Five-year follow-up of a randomized controlled trial. Ann Surg.

[6] Somashekhar SP, Gupta P, Ballal S, Parameshwaran, Zaveri SS, Venkatachala (2007). Minimally invasive radioguided surgery for parathyroid adenomas (MIRP). Natl Med J India.

[7] Caudle AS, Brier SE, Calvo BF, Kim HJ, Meyers MO, Ollila DW (2006). Experienced radio-guided surgery teams can successfully perform minimally invasive radio-guided parathyroidectomy without intraoperative parathyroid hormone assays. Am Surg.

[8] Lo Gerfo P (1999). Bilateral neck exploration for parathyroidectomy under local anesthesia: A viable technique for patients with coexisting thyroid disease with or without sestamibi scanning. Surgery.

[9] Costello D, Norman J (1999). Minimally invasive radioguided parathyroidectomy. Surg Oncol Clin N Am.

[10] Kuriloff DB, Sanborn KV (2004). Rapid intraoperative localization of parathyroid glands utilizing methylene blue infusion. Otolaryngol Head Neck Surg.

[11] Blocklet D, Martin P, Schoutens A, Verhas M, Hooghe L, Kinnaert P (1997). Presurgical localization of abnormal parathyroid glands using a single injection of technetium-99m methoxyisobutylisonitrile: Comparison of different techniques including factor analysis of dynamic structures. Eur J Nucl Med.

[12] Moure D, Larrañaga E, Domínguez-Gadea L, Luque-Ramírez M, Nattero L, Gómez-Pan A (2008). 99MTc-sestamibi as sole technique in selection of primary hyperparathyroidism patients for unilateral neck exploration. Surgery.

[13] Mullan BP (2004). Nuclear medicine imaging of the parathyroid. Otolaryngol Clin North Am.

[14] Bugis SP, Berno E, Rusnak CH, Chu D (1995). Technetium99m-sestamibi scanning before initial neck exploration in patients with primary hyperparathyroidism. Eur Arch Otorhinolaryngol.

[15] Farley DR (2004). Technetium-99m 2-methoxyisobutyl isonitrile-scintigraphy: Preoperative and intraoperative guidance for primary hyperparathyroidism. World J Surg.

[16] Merlino JI, Ko K, Minotti A, McHenry CR (2003). The false negative technetium-99m-sestamibi scan in patients with primary hyperparathyroidism: Correlation with clinical factors and operative findings. Am Surg.

[17] Erbil Y, Kapran Y, Işsever H, Barbaros U, Adalet I, Dizdaroğlu F (2008). The positive effect of adenoma weight and oxyphil cell content on preoperative localization with 99mTc-sestamibi scanning for primary hyperparathyroidism. Am J Surg.

[18] Liechty RD, Teter A, Suba EJ (1986). The tiny parathyroid adenoma. Surgery.

[19] Erbil Y, Barbaros U, Yanik BT, Salmaslioğlu A, Tunaci M, Adalet I (2006). Impact of gland morphology and concomitant thyroid nodules on preoperative localization of parathyroid adenomas. Laryngoscope.

[20] Stephen AE, Roth SI, Fardo DW, Finkelstein DM, Randolph GW, Gaz RD (2007). Predictors of an accurate preoperative sestamibi scan for single-gland parathyroid adenomas. Arch Surg.

